# Effect of Graphene vs. Reduced Graphene Oxide in Gold Nanoparticles for Optical Biosensors—A Comparative Study

**DOI:** 10.3390/bios12030163

**Published:** 2022-03-04

**Authors:** Ana P. G. Carvalho, Elisabete C. B. A. Alegria, Alessandro Fantoni, Ana M. Ferraria, Ana M. Botelho do Rego, Ana P. C. Ribeiro

**Affiliations:** 1 Departamento de Engenharia Química, ISEL, Instituto Politécnico de Lisboa, 1949-014 Lisbon, Portugal; elisabete.alegria@isel.pt; 2 Centro de Química Estrutural, Instituto Superior Técnico, Universidade de Lisboa, 1049-001 Lisbon, Portugal; apribeiro@tecnico.ulisboa.pt; 3 Departamento de Engenharia Eletrónica e Telecomunicações e de Computadores, ISEL, Instituto Politécnico de Lisboa, 1949-014 Lisbon, Portugal; afantoni@deetc.isel.ipl.pt; 4 Centro de Tecnologias e Sistemas, UNINOVA, Faculdade de Ciências e Tecnologia, 2829-517 Caparica, Portugal; 5 iBB—Institute for Bioengineering and Biosciences and Departamento de Engenharia Química, Instituto Superior Técnico, Universidade de Lisboa, Av. Rovisco Pais, 1049-001 Lisbon, Portugal; ana.ferraria@tecnico.ulisboa.pt (A.M.F.); amrego@tecnico.ulisboa.pt (A.M.B.d.R.); 6 Associate Laboratory i4HB—Institute for Health and Bioeconomy at Instituto Superior Técnico, Universidade de Lisboa, Av. Rovisco Pais, 1049-001 Lisbon, Portugal

**Keywords:** biosensors, AuNPs, metal–graphene hybrid, simulations, Mie theory

## Abstract

Aiming to develop a nanoparticle-based optical biosensor using gold nanoparticles (AuNPs) synthesized using green methods and supported by carbon-based nanomaterials, we studied the role of carbon derivatives in promoting AuNPs localized surface plasmon resonance (LSPR), as well as their morphology, dispersion, and stability. Carbon derivatives are expected to work as immobilization platforms for AuNPs, improving their analytical performance. Gold nanoparticles (AuNPs) were prepared using an eco-friendly approach in a single step by reduction of HAuCl_4_·3H_2_O using phytochemicals (from tea) which act as both reducing and capping agents. UV–Vis spectroscopy, transmission electron microscopy (TEM), zeta potential (ζ-potential), and X-ray photoelectron spectroscopy (XPS) were used to characterize the AuNPs and nanocomposites. The addition of reduced graphene oxide (rGO) resulted in greater dispersion of AuNPs on the rGO surface compared with carbon-based nanomaterials used as a support. Differences in morphology due to the nature of the carbon support were observed and are discussed here. AuNPs/rGO seem to be the most promising candidates for the development of LSPR biosensors among the three composites we studied (AuNPs/G, AuNPs/GO, and AuNPs/rGO). Simulations based on the Mie scattering theory have been used to outline the effect of the phytochemicals on LSPR, showing that when the presence of the residuals is limited to the formation of a thin capping layer, the quality of the plasmonic resonance is not affected. A further discussion of the application framework is presented.

## 1. Introduction

Plasmonic biosensors are widely explored as promising sensing tools due to their low cost, simplicity, and short response time. These devices can be useful in a variety of situations, such as medical emergencies in more isolated populations or/and with poor access to health services [1]. For these applications, plasmonic structures can be integrated in point-of-care systems with optical, electrical, or thermal signals delivered in response to certain stimuli, mediated by biomolecules immobilized on biosensor surfaces—biological recognition elements (BREs)—thereby allowing the selective detection of analytes of interest [2].

Gold nanoparticles (AuNPs) have been widely used in the development of optical biosensors [3]. Their utilization in optical signal analysis is based on their characteristic surface plasmon resonance (SPR) effects. No additional material is required for the generation of surface plasmons after interaction with light [4], making their use advantageous in label-free sensing devices.

When AuNPs are used as optical transducers, the region of confinement in which the evanescent wave is propagated is smaller than the wavelength of the incident light. In this case, the phenomenon is called localized surface plasmon resonance (LSPR), where the collective oscillation of the electrons originates a dipolar or multipolar moment in the nanoparticle. LSPR is excited at a specific light wavelength, which is determined by the morphology, size, and composition of the nanoparticle [5]. Any modification on the surface of AuNPs affects the behavior of the LSPR, allowing the exploration of this phenomenon in the development of biosensors through the functionalization of its surface with biomolecules.

When the analyte of interest is recognized by the BRE, the surface refractive index of the AuNPs changes, promoting a shift in the LSPR wavelength [4].

The interest in AuNPs for the development of biomedical diagnostic devices is related to their sensitivity and the possibility of controlling and optimizing the limit of detection (LOD) [5].

Moreover, these interesting properties may act synergistically with graphene when this carbon nanomaterial is added to form a nanocomposite [6]. For example, Banerjee [7] highlighted the efficiency of nanocomposites formed with metallic nanoparticles and graphene in biomedical applications as compared to conventional materials. This greater efficiency is due to their small size and high surface-to-volume ratio, which improves their responses to external stimuli. Carbon allotropes, such as graphene (G), graphene oxide (GO), and reduced graphene oxide (rGO), have similar optical, electronic, and electrochemical properties, and have therefore been investigated as potential materials for biosensor design, drug delivery, bioimaging, and tissue engineering applications. The unique properties of graphene derivatives are also related to their 2D dimensionality, high electrical conductivity and thermal properties, malleability, and functionalization potential. The properties of graphene, which is composed of a single 2D sheet of carbon atoms forming vertices of hexagons with covalent bonds and *sp^2^* hybridization [7], can be manipulated by chemical modification either through oxidation (from G to GO) or reduction (from GO to rGO) reactions, increasing its functionalization capabilities and broadening its range of applications.

The oxidation of graphene, yielding graphene oxide (GO), allows the addition of oxygen functional groups to its surface, reducing electrical conductivity and malleability, and increasing solubility in polar solvents, as well as reducing the aggregation of carbon-based nanomaterials in aqueous solutions.

Graphene oxide shows a greater functionalization capacity due to the presence of a larger number of oxygen groups, but for the same reason there is also restriction of the mobility of electrons on its surface. Reduction of the number of oxygen groups, yielding reduced graphene oxide, allows the restructuring of electrical and thermal conductivity towards pristine graphene, maintaining functionalization capacity and the distance between graphene sheets [7]. The maintenance of this distance is mandatory to prevent agglomeration between the sheets and avoid the graphite state, since this material has different properties to graphite-derivative nanomaterials [8]. The AuNPs act synergistically with these nanoscale carbon-based nanomaterials, not only in optical properties but also in the dispersion of both nanoparticles and graphene sheets. Studies report that nanoparticles can function as nano-spacers of these materials [9], enhancing AuNPs’ stability after the addition of rGO.

In this work, the influence of a variety of carbon-based materials on gold nanoparticles’ (AuNPs) localized surface plasmon resonance (LSPR) has been studied and, considering environmental concerns, an ecofriendly approach in the synthesis of gold nanoparticles was chosen, using tea extract as a reducing/capping agent. As a matter of fact, the phytochemicals present in tea, such as polyphenols and tannins, have the interesting capacity of reducing metallic salts, such as chloroauric acid (HAuCl_4_) [10,11,12].

Although the use of composite materials based on metal nanoparticles and graphene allotropes to explore plasmonic properties in sensing applications has already been reported in the literature [13,14], a green method of synthesis for nanoparticle fabrication represents a novel approach in the preparation of hybrid materials. Since a residual layer of polyphenols remains on the surface of the nanoparticles, this approach results in the introduction of an additional factor in the set of parameters necessary to obtain the desired LSPR tuning which deserves to be investigated. In this paper, the frequency and intensity of the LSPRs for three different types of graphene are analyzed, compared, and discussed, combined with a green protocol for the synthesis of gold nanoparticles as well as different sequences in the fabrication of the composites.

### Application Framework

Sensors based on the local surface plasmon resonance of metal nanoparticles are characterized by simple structures and good sensitivities. Despite the good sensing properties of LSPR structures, full commercialization has been prevented by the production costs associated with the bio-functionalization and the high-precision systems necessary to extract the optoelectronic output. There is a great interest in new strategies for bringing the excellent detection properties of LSPR sensors into play in low-cost devices made with low-cost materials [15]. The combination of carbon-based nanomaterials (CNMs) with MNPs has been demonstrated to enhance LSPR response [16] and facilitate functionalization with specific and selective antibodies [17]. In addition, the introduction of CNMs in the plasmonic layer allows a tuning of the LSPR central frequency. With the double dependence of the LSPR on MNP size and the presence of CNMs, it is possible to create a set of plasmonic layers whose LSPR wavelengths are distributed in a spectral range of a few tenths of a nanometer. This consideration paves the way to an LSPR sensor with an arrayed structure, where each element maximizes its specific LSPR at its own wavelength. Illumination with a broad light source will produce a different response in each one of the elements and the biomarkers’ immobilization in the surrounding medium will cause a transition to a different state. In such a configuration, the output can be extracted by the application of an image analysis approach based on a color-recognition algorithm [18]. The experimental characterization presented in this work represents a first step toward the development of an arrayed LSPR sensor whose elements are composed by metal nanoparticles with different dimensions and supported by different CNMs, joined to a reading scheme provided by a CCD imager, supported by an image processing algorithm. The use of low-cost materials together with a simplified interrogation scheme aims to overcome the elevated costs related to high-precision mechanical systems and wavelength selective light sources. The result will be a proof of concept for a low-cost LSPR sensor with potential for large-scale biosensing applications in environmental monitoring or the medical stratification of diseases.

The main advantages of these nanomaterials are their biocompatibility, high chemical stability, and high surface areas. The reduced dimensions and weight, high resistance, simplicity of use, and low cost makes graphene and its derivatives excellent candidates for the development of biosensors.

An optical sensor has, by definition, the ability to convert an external stimulus into an optical output signal.

The π–π binding capacity of graphene with biomolecules allows the use of this carbon-derived nanomaterial as a substrate, and the SPR technique can be used to detect the interaction with the analyte of interest, in which the angle of an incident polarized light is adjusted as a result of the change in the refractive index caused by the interaction between them. rGO has advantages over graphene, since its oxygen functional groups improve the interaction of this nanomaterial with biomolecules [19].

Optical sensors, such as those based on fluorescence, surface-enhanced Raman scattering (SERS), optical fiber biological sensors, among other kinds of optical sensors, are being developed due to the amazing properties of graphene and its derivatives. Various sensing applications, such as single-cell detection, cancer diagnosis, and protein and DNA sensing, have been reported in recent years [19].

Due to its unique optical and electrical properties, graphene and its derivatives are widely used in photonic and optoelectronic devices as they have displayed several ideal properties, including broadband light absorption, the ability to quench fluorescence, excellent biocompatibility, and strong polarization-dependent effects, making them very popular platforms for optical sensors. Graphene and its derivatives-based optical sensors have numerous advantages, such as high sensitivity, low-cost, fast response time, and small dimensions. The use of metallic nanoparticles, namely, gold nanoparticles, may improve the response of biosensors, amplifying the signals obtained and increasing the sensitivity of these devices [19].

Several applications of graphene and its derivatives-based optical sensors are summarized in the following Table 1:

It is undeniable that there are still many challenges in this area. The need to synthesize high-quality graphene, to achieve a low-cost, environmentally friendly method for synthesizing graphene and its derivatives are issues still to be addressed. With this study, the use of a simple green method to produce light-responsive material is our aim and contribution.

Addressing the above challenges, we hope to show the potential of green methods as well as the importance of graphene and its derivatives in the development of optical sensing technologies, which will ultimately increase the quality of future life.

## 2. Results and Discussion

### 2.1. Sequence 1—Addition of Carbon-Based Derivatives to AuNPs (SQ1)

**Figure 1:**
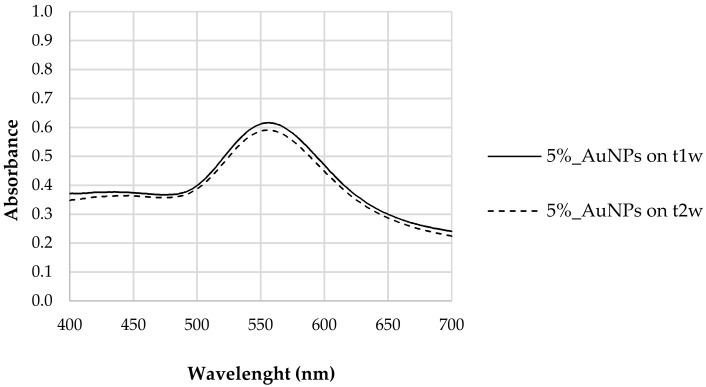
LSPR of AuNP samples synthesized with 5% tea extract (*Thea sinensis*) at t_1w_ (after 1 week) and t_2w_ (after 2 weeks).

After the addition of chloroauric acid (HAuCl_4_·3H_2_O) to a tea extract (5% *w*/*w*), a change of color from yellow to red was observed, which is consistent with the formation of AuNPs. The AuNPs’ characteristic LSPR was observed only after one week (t_1w_), the absence of LSPR at the initial time (t_0_) possibly being associated with the period of reduction required to originate AuNPs or due to the fact that in the case of hybrid nanostructures, carbon materials may be responsible for radiation absorption, inhibiting the detection of AuNPs, as described by Biris et al. [37]. Figure 1 shows the UV–Vis spectrum of the aqueous solution containing AuNPs with the corresponding LSPR occurring at 556 nm. The presence of a single band suggests a spherical morphology for the synthesized AuNPs [37]. The solution remained stable for several weeks without any signal of aggregation.

The stability of the synthesized nanoparticles was evaluated. We found that between the first (t_1w_) and second week (t_2w_) after synthesis no significant differences in LSPR response was observed, confirming that polyphenols guarantee a certain stability to the synthesized AuNPs. As proven previously by the authors of [10], polyphenols prevailed as reducing agents of the metallic salt and capping agents of the produced AuNPs [10], maintaining their capacity for at least two weeks after the synthesis. This is due to the ionic force of polyphenols that promote good dispersion by efficiently counteracting the mutual attraction caused by Van der Waals forces and which are responsible for the aggregation of nanoparticles [38].

**Figure 2:**
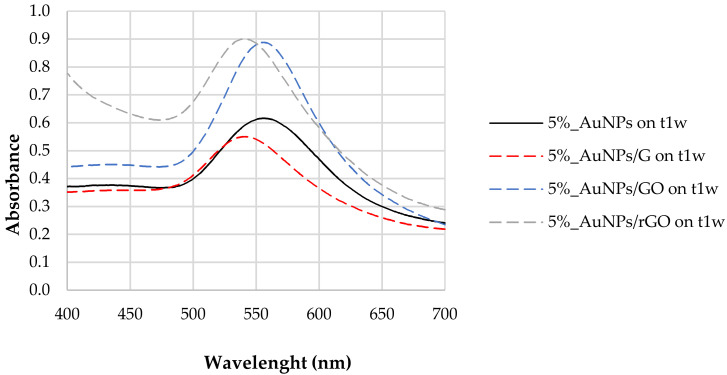
LSPR of AuNPs synthesized with 5% tea (*w*/*w*) extract and after the addition of G, GO, and rGO, one week after their synthesis (t_1w_).

Figure 2 reports the LSPR of AuNPs synthesized with 5% tea extract after the addition of G, GO, and rGO. For the naked AuNPs (5%_AuNPs), the LSPR at t_1w_ occurred at 556 nm, with a shift to higher frequencies for the mixtures containing graphene (G) (LSPR = 541 nm) and reduced graphene oxide (rGO) (LSPR = 542 nm). The composite containing graphene oxide (AuNPs/GO) did not show any LSPR shift after the addition of carbon-based nanomaterials. For the AuNPs/G hybrid nanostructure, band amplitude decreased by ~10% when compared to the sample without any of the carbon-based nanomaterial, while for AuNPs to AuNPs/rGO, it increased by ~0.29, which corresponds to 46% of the naked/unsupported AuNPs’ absorbance. This result may be ascribed to a dispersion of the AuNPs [38] in the presence of the polyphenols [37].

**Figure 3:**
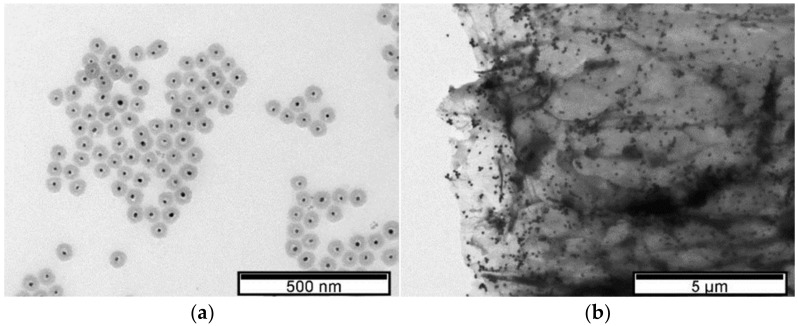
TEM images. (**a**) AuNPs in 5% tea extract. (**b**) rGO-supported AuNPs in 5% tea extract.

LSPR properties depend not only on the AuNPs’ shape, size, and dispersion but also on the surrounding dielectric constant. TEM characterization showed the formation of spherical AuNPs (Figure 3). Although the addition of rGO caused a LSPR shift to lower wavelengths, the AuNPs supported on rGO exhibited a larger diameter (61 nm ± 3 nm) when compared to AuNPs only encapsulated by polyphenols (30 nm ± 2 nm). The shift to lower wavelengths after the carbon-based material addition may be related to the increased dispersion of AuNPs, with no change in the diameter of the nanoparticles, and to charge transfer interactions between graphene and AuNPs [39]. By acting as nano-spacers [6], when graphene is used as a support, the nanoparticles benefit from increased dispersion, while at the same time they avoid graphene sheet agglomeration. Additionally, the LSPR deviation can also be caused by the surface energy of neighboring nanoparticles [40].

The probable formation of π–π bonds between AuNPs and the functional oxygen groups of rGO [6] may be responsible for the stable dispersion confirmed by the measured zeta potential value −20.17 mV when compared to −15.59 mV obtained for the sample containing AuNPs dispersed in 5% tea extract (Table 2). Since LSPR did not occur at t_0_, we assumed that the nucleation of metallic salts occurred near the rGO functional oxygen groups [6] which may have promoted a higher stability due to the dispersion of the AuNPs as well as the increase in the diameter of the nanoparticles [41].

**Figure 4:**
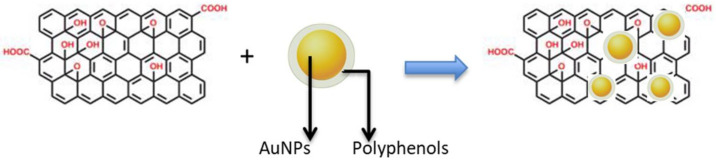
Schematic illustration of AuNPs anchored on the surface of reduced graphene oxide (rGO).

The reason for using a pre-prepared solution of AuNPs stabilized with polyphenols then mixed with rGO is to help anchor the polyphenol-protected AuNPs to the rGO surface. This process could be assisted by: (i) *π*–*π* interactions between AuNPs and functional oxygen groups of rGO; (ii) *π*–*π* interactions between benzene rings of the polyphenols and the surface of rGO; and (iii) electrostatic interactions between the OH on the surface of rGO and the polyphenols capping the AuNPs. After mixing, the shape of the AuNPs appeared to be the same when observed by TEM (Figure 3), but the presence of the rGO seemed to have anchored them, as expected. Schematically (Figure 4), the introduction of rGO leads to the settling of the AuNPs [42].

**Figure 5:**
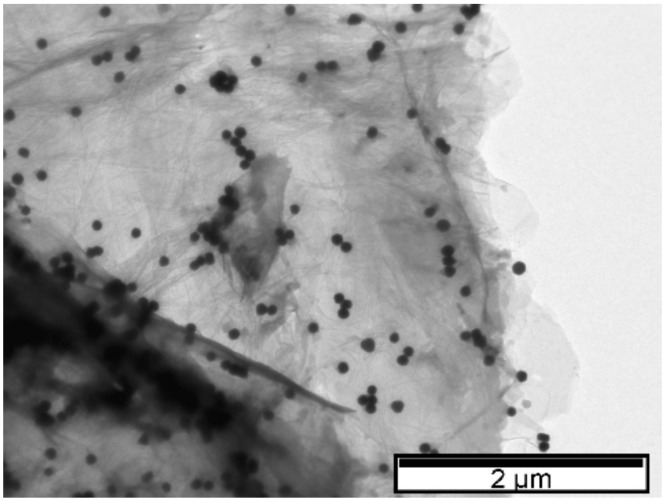
TEM image. GO supported on AuNPs synthesized with 5% tea extract.

In the case where GO is added to the AuNPs, no significant deviation in the LSPR occurs relative to the value observed prior to the addition of this carbon-based material (λ = 556 nm). TEM images (Figure 3) show a greater dispersion of AuNPs on the surface of the carbon-based nanomaterial which is probably responsible for the increase in the LSPR band amplitude observed by UV–Vis [38,43,44]. As with the addition of rGO, nucleation may have occurred next to the functional oxygen groups [41]. To confirm this hypothesis, Figure 5 shows dispersed nanoparticle clusters on the GO surface. The wide number of oxygen functional groups on the GO may have promoted AuNP agglomeration and significant dispersion of diameters. These results are in accordance with those reported by Parnianchi et al. [45] related to the difficulty of controlling both the morphology and the homogeneous distribution of AuNPs when GO is present. The frequency versus size distributions for the three TEM images are presented in ESI (Figure S1).

After the addition of both GO and rGO, sharper resonance bands as well as an increase in the resonance intensity or amplitude were observed (Figure 2). In fact, both enhancements of plasmonic resonance intensity and shift in the position are indicators of enhanced sensitivity of the LSPR sensor.

The partial reduction of oxygen groups in GO driven by tea polyphenols [46] may contribute to reducing the availability of these phytochemicals both as reduction and capping agents. The dispersion of diameters verified after GO addition and the fact that this nanomaterial is insulating (due to the functional groups of oxygen, the electronic mobility is reduced) compromises the use of this nanocomposite in the development of biosensors.

We also measured the electrostatic potential at the electrical double layer surrounding a nanoparticle in solution, commonly referred to as the zeta potential. Nanoparticles with a zeta potential between −10 and +10 mV are considered approximately neutral, while nanoparticles with zeta potentials of greater than +30 mV or less than −30 mV are considered strongly cationic and strongly anionic, respectively [47]. Since most cellular membranes are negatively charged, zeta potential can affect the nanoparticle tendency to permeate membranes, with cationic particles generally displaying more toxicity associated with cell wall disruption. It can be observed that the initial 5%_AuNPs are weakly anionic. With the addition of rGO, they became strongly anionic due, most likely, to the increase in acidity by the introduction of rGO.

According to the classical physical theory for the electromagnetics of metals, the plasma frequency (*ω_p_*) of the free electron gas depends linearly on the density of the electrons (N_e_). Such a consideration leads, in the Drude model, to a linear dependence of the metal dielectric function on the value of N_e_. Within the dipole approximation, i.e., when the nanoparticle size is much smaller than the light wavelength, and under the Fröhlich condition, it can be shown that the LSPR frequency (*ω_LSPR_*) can be directly related to *ω_p_* by the following equation:

$$\omega_{LSPR}=\frac{\omega_{p}}{\sqrt{1+2\varepsilon_{m}}}\;$$

where *ε_m_* is the dielectric constant of the surrounding medium [48]. Therefore, a change in the electron density is expected, leading to a blue or red shift of the localized plasmon resonance. This effect has been reported in the literature, supported by electrochemical experiments [49] and exploited for sensing applications [38]. Optical gas sensors based on gold nanoparticles and carbon nanomaterials have also been demonstrated to rely on the reactions for both reducing and oxidizing gases and the correspondent injection or subtraction of electrons to and from graphene oxide, implying a shift in the observed LSPR [50]. The charge transfer interaction between AuNPs and graphene has been also demonstrated to be suitable for active modulation of surface plasmon resonance. These considerations are in agreement with our experimental findings, namely, the blue shift observed in the LSPR of the composites (Figure 2) and the negative charge transfer between the graphene allotropes and the nanoparticles observed in the zeta potential measurements (Table 2).

The degree of oxidation of graphene before and after the addition of AuNPs, the type of oxygen functional groups, the oxidation state of gold at AuNP surfaces within the AuNPs/G, AuNPs/GO, or AuNPs/rGO composites, as well as relevant relative atomic amounts were assessed by XPS. The characterization of AuNPs prior to any graphene addition has already been reported [10]. Here, the three different types of graphene and the corresponding gold composites are studied.

XPS confirms that samples are composed mainly of carbon and oxygen, and, where expected, gold. GO-based samples also contain some sulphur, and some samples have a residual or low relative amount of silicon. Table 3 shows the corrected binding energies (BE) of the peaks fitted in the different XPS regions: C 1s, O 1s, Au 4f, S 2p, and Si 2p.

**Figure 6:**
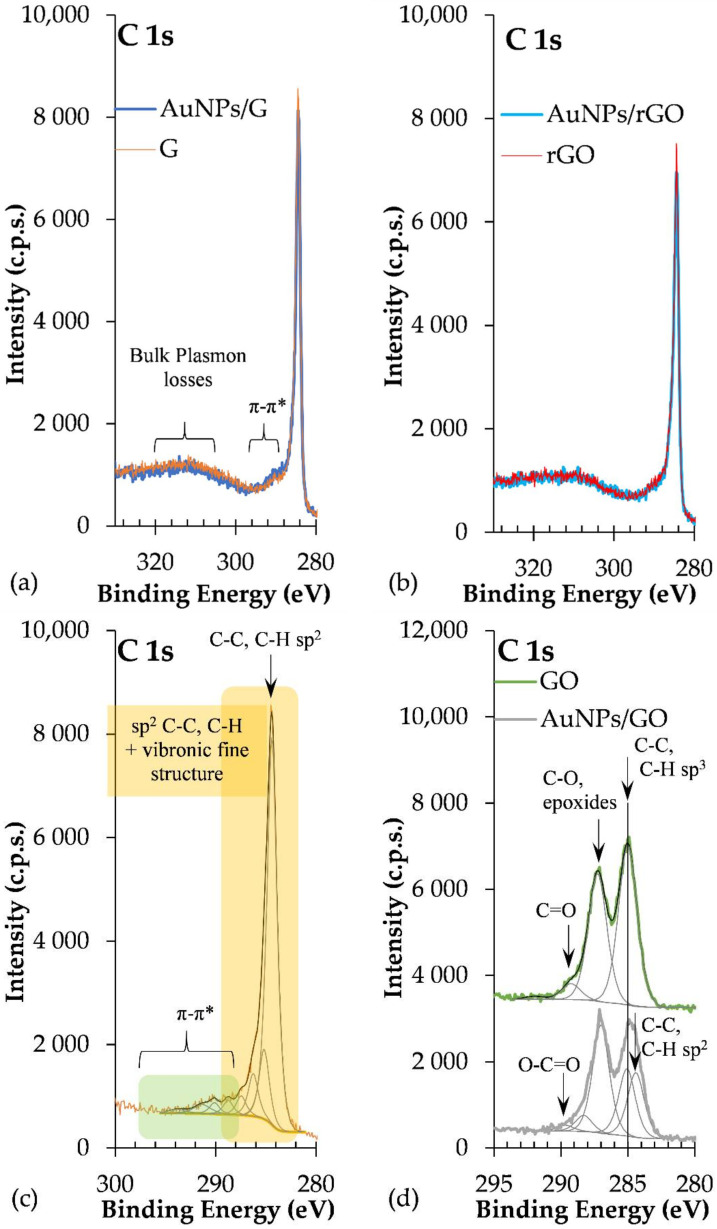
C 1s regions of (**a**) AuNPs/G and G; (**b**) AuNPs/rGO and rGO; (**c**) G with fitting (similar to rGO and AuNPs/rGO and AuNPs/G); and (**d**) GO and AuNPs/GO.

C 1s regions of AuNPs/rGO and AuNPs/G are dominated by the peak assigned to carbon atoms in C-C and C-H sp^2^ bonds, centred at 284.4 ± 0.1 eV, and by a long tail at the high BE side, detected roughly between 287 eV and 297 eV (Figure 6a–c), corresponding to π–π* excitations, typical of extended delocalized systems, such as graphene. In addition, at BE > 297 eV plasmon loss features are detected. Included in the C 1s envelope, peaks attributed to carbon atoms bonded to oxygen in different functional groups are identified in Table 3. The latter are particularly intense in GO and AuNPs/GO, where the loss of electron delocalization due to the oxidation of graphene is evident: the sp^2^ carbon peak has a lower relative intensity compared with AuNPs/rGO and AuNPs/G, and the energy loss features are hardly detected (Figure 6d). In GO, a peak at 284.4 eV was fitted, but it is almost cancelled with the fitting, leaving only the peak centred at 285 eV (Figure 6d).

**Figure 7:**
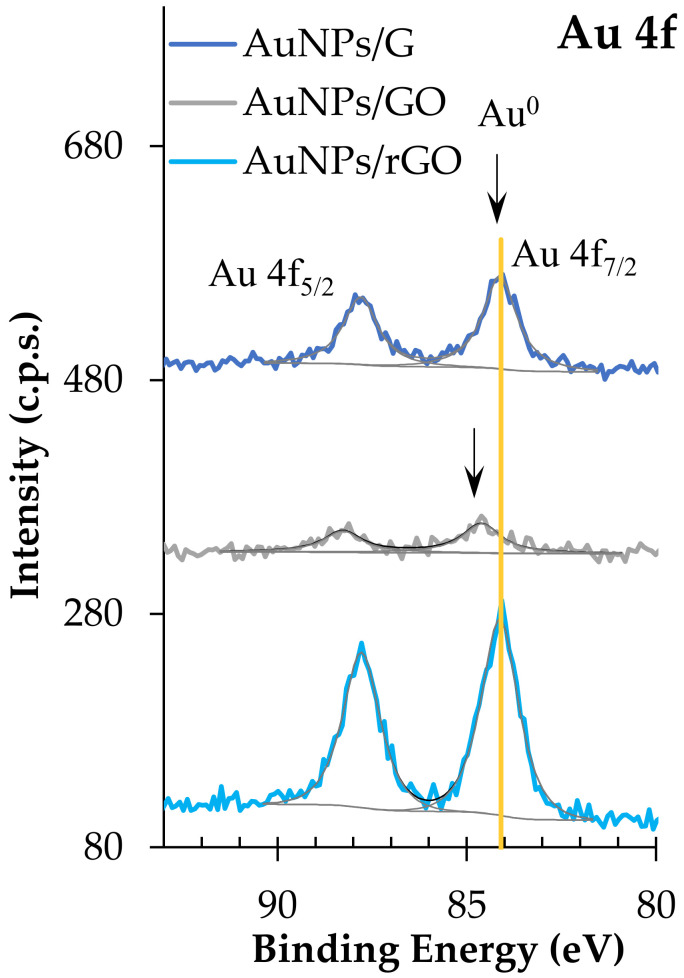
Au 4f XPS regions.

Au 4f regions are doublet peaks (Figure 7) with a spin–orbit energy separation of 3.7 eV. Au 4f_7/2_ is centred at 84.1 ± 0.1 eV in AuNPs/rGO and AuNPs/G, which is attributed to Au^0^. In AuNPs/GO, Au 4f_7/2_ is centred at 84.6 ± 0.1 eV, which has been identified as Au^+^ in HAuCl [53]. However, in this case, no chlorine was detected to corroborate this assignment. Still, positive BE shifts for Au 4f photoelectrons have been reported for very small gold nanoparticles (diameter ≤20 nm) [54]. In addition, a contact potential effect between the metal nanoparticles and the organic substrate may be present, leading to an underestimation of the charge shift. It is noteworthy that the full widths at half maximum, for all Au 4f fitted peaks, are very similar to each other (1.1 ± 0.1 eV), which also sustains the hypothesis of having reduced gold nanoparticles in all the samples. Finally, sulphur is only present in GO-based samples. S 2p is a doublet with a spin–orbit split of 1.1 ± 0.1 eV, and the main component, S 2p_3/2_, centred at 168.8 ± 0.2 eV, is assigned to sulphate groups. The silicon detected in some of the samples may come from the polysiloxane-based tape used to mount the powder for XPS analysis. Table 4 shows the XPS quantitative analysis.

It is interesting to note that the larger relative amount of Au in AuNPs/rGO compared with AuNPs/G, computed from XPS data, is compatible with the UV–Vis absorbance spectra shown in Figure 2: AuNP LSPR absorbance is much larger for AuNPs/rGO than for AuNPs/G. Moreover, the relative amount of Au, detected by XPS, is much lower in AuNPs/GO than in AuNPs/rGO. Actually, as attested by Table 3 and Figure 6, graphene oxide has a very different chemical composition from G or rGO, with many more oxygen functional groups than G or rGO. These oxidized carbonaceous groups establish stronger intermolecular interactions with polyphenols surrounding the AuNPs, allowing for the formation of sandwich-like GO/AuNPs/GO, significantly attenuating the Au 4f photoelectron signal detected by XPS. It is also clear from the quantification results that samples with gold nanoparticles are slightly more oxidized than the parent samples with no gold. Actually, since AuNPs are capped with phenolic functional groups, a larger relative amount of oxygen is expected in the samples with AuNPs. In addition, a further reduction of gold may occur when in contact with G, GO, or rGO with the simultaneous oxidation of graphene. In Table 4, the ratios computed were obtained after subtracting the contribution of sulphate and silicone, these being, exclusively, the ratios in the graphene-based samples discarding the contaminations. Other effects of the introduction of Au nanoparticles can be found in the C 1s spectral differences presented in ESI (Figure S2).

### 2.2. Sequence 2—Addition of Carbon-Based Derivatives Prior to AuNP Formation (SQ2)

The addition of carbon-based nanomaterials prior to AuNP formation seems to compromise phytochemicals’ reducing and capping capacities. This may be related to the adsorption of phytochemicals by carbon-based nanomaterials [6] which compromise their availability to reduce the metallic salt. Some of these samples revealed a slight LSPR at t_0_ (Figures S4–S6, Supplementary Materials). We believe that the addition of these derivatives initially increases the contact between the metallic salt and polyphenols, promoting the synthesis of AuNPs. However, at t_1w_ and t_2w_ the LSPR did not occur. We believe that the adsorption of polyphenols by carbon-based nanomaterials through electrostatic bonds and Van der Waals interactions [6] compromises the efficiency of these species as capping agents. In this case, we did not verify stability between t_1w_ and t_2w_ in any of the samples. No further testing was performed.

### 2.3. AuNP Stability Study

**Figure 8:**
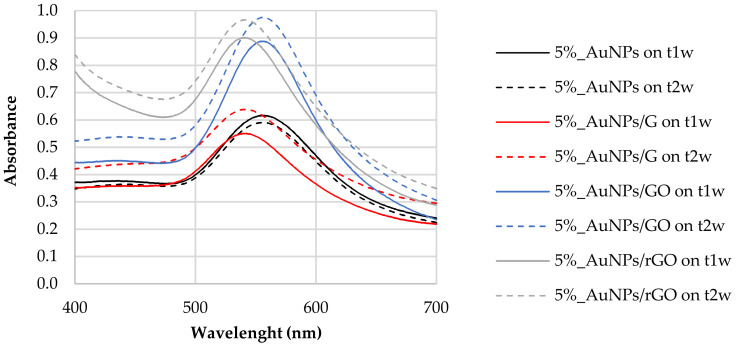
AuNP samples stability (t_1w_–t_2w_). Carbon-based nanomaterials added after AuNP formation.

The stability of the synthetized nanoparticles was also evaluated by the sample’s characterization, 1 week (t_1w_) and 2 weeks (t_2w_) after synthesis. Figure 8 reports the absorbance spectra for the different composites taken at time intervals of one week. We found that the resonance of the surface plasma had a red shift one week after synthesis (t_1w_ vs. t_0_)_._ These data show that the synthesis of the nanoparticles did not cease after 20 min of vigorous stirring of chloroauric acid with the tea extract. This shift could mean that the diameters of the AuNPs increased between t_0_ and t_1w._ As mentioned above, the increased band amplitude after a week suggests that there was an increase in the concentration of nanoparticles, indicating that the formation of AuNPs was still happening [44].

UV–Vis analysis of the samples after 2 weeks (t_2w_) showed that LSPR did not shift in comparison to that observed after 1 week (t_1w_) (Figure 8). The polyphenols acted as capping agents [10] of the AuNPs contrary to the Van der Waals forces that promote the agglomeration of AuNPs. This result leads us to conclude that polyphenols can be good candidates for green synthesis of AuNPs, not only because they were efficient as reducing agents but also because they gave stability to synthesized nanoparticles. The synthesis with tea extract allows the use of phytochemicals for the production and stabilization of AuNPs, simplifying the process.

Between t_1w_ and t_2w_, we can observe a slight increase in the absorbance value without a change in the behavior of the plasmonic response. Tea contains a multitude of different chemicals. Some of these, e.g., tannins, are fairly dark to begin with, but, if they are allowed to react with the oxygen [55] in the air, they oxidize, producing other compounds that are even darker in color. We can observe this phenomenon by UV spectroscopy because, in general, bigger molecules absorb more light, and as the oxidized tannins tend to aggregate over time [56], creating bigger molecules, this leads to a change in tea spectra. GO and rGO oxygen functional groups contribute to a more efficient oxidation of samples. The difference between t_1w_ and t_2w_ is more obvious in the 5%_AuNPs/GO in comparison with the 5%_AuNPs/rGO sample, since GO has more oxygen groups on its surface.

### 2.4. Simulation (Mie Theory)

The variation in the transmission spectra caused by the plasmonic resonance of the nanoparticles can be calculated by recourse to the Mie Theory [57], and the intensity of the LSPR effect can be correlated with the material properties of the surrounding medium [58]. Regarding gold spherical nanoparticles, as a bottom line of the Mie analysis, it can be stated, as a rule, that increasing AuNP size results in an LSPR shift towards the red part of the spectrum. Such a behavior can also be observed in experimental measurements [59]. Moreover, as the ratio between AuNP radius and light wavelength increases, multipolar behavior is to be expected and a widening of the peak waist observed. Increasing the values of the refractive index of the surrounding medium will also lead to a red shift of the LSPR peak, accompanied by a significant enhancement of its maximum value. For the analysis of the specific case of the AuNPs, produced by combining HAuCl_4_ with the phytochemicals present in tea extract as a reducing agent, we have considered the gold nanosphere capped by a thin uniform layer of tea polyphenols, immersed in pure water. This approach agrees with the morphology observed in Figure 3a. The most abundant polyphenol encountered in tea is epigallocatechin gallate (EGCG) [60], whose reported refractive index is 1.857 [61], which was the value used in the simulations.

**Figure 9:**
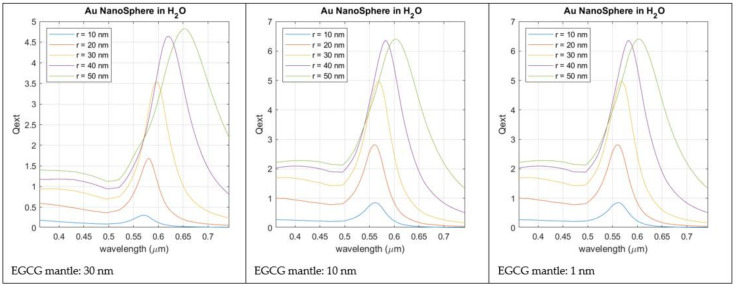
Simulated LSPR intensity for AuNPs with increasing dimensions (radius between 10 and 50 nm). Gold nanospheres are immersed in pure water and have a capping layer of EGCG with a thickness between 1 and 30 nm.

Figure 9 reports the light extinction profile calculated for a AuNP with increasing radius and capped by a thin layer of EGCG (1–30 nm). From the analysis of this Figure, the LSPR wavelength (560 nm for a 10 nm radius of the AuNP) is only slightly affected by the thickness of the EGCG layer, but if it is too thick, LSPR intensity is reduced. Anyway, when compared with the LSPR produced by AuNPs in pure water, without EGCG capping, the LSPR peak always suffers a red shift. This red shift is observable for any thickness of the capping layer.

**Figure 10:**
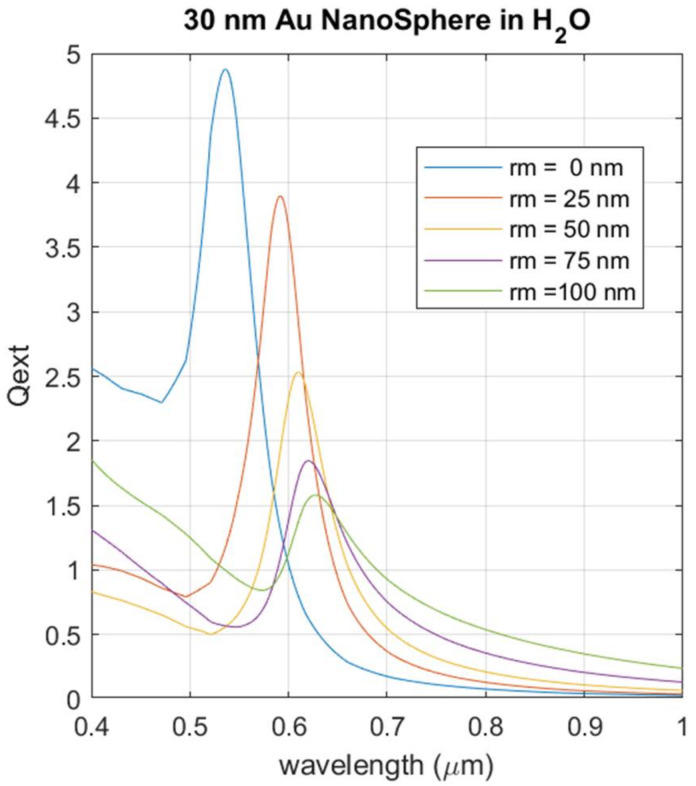
Simulated LSPR intensity for AuNPs with fixed dimensions (radius 30 nm). Gold nanospheres are immersed in pure water and have a capping layer of EGCG with thickness between 0 and 100 nm.

Figure 10 shows the results for the simulation where the light extinction is calculated for a AuNP with a radius of 30 nm and a capping layer thickness between 1 and 100 nm.

The effect of increasing the thickness of the cover layer results in the LSPR shifting towards the red region. Simultaneously, the intensity of the LSPR shows a marked reduction as the capping thickness increases.

We can conclude from this simulation analysis that the residual polyphenols which remain after AuNP fabrication have a negligible effect on the quality of the plasmonic response. An excessive accumulation of the residual polyphenols on the AuNP surface would reduce the LSPR intensity, but the simulation shows that for the range of the capping thickness observed in the TEM images, such a level of EGCD accumulation is not reached. The red shift foreseen by the simulation has no impact on the operation mechanism of a sensor device built with these materials. At the same time, the presence of the capping layer can be expected to physically separate the nanoparticles, preventing their aggregation. Thus, AuNPs synthesized by this green method combine the advantage of a simplified fabrication method that avoids aggregation with a reliable plasmonic resonance that can be successfully exploited in a sensing device.

The wavelength and intensity of LSPR are strongly dependent on the refractive index of the surrounding medium. An alteration of this parameter provokes a shift of the LSPR peak which can be used as the output value of a sensing system. To evaluate the sensing efficiency of the AuNPs, the spectral shift (Δλ) of the resonance wavelength, as a function of the variation in the refractive index (Δn) of the surrounding medium, can be translated into a sensitivity parameter (S):

S = Δλ/Δn

**Figure 11:**
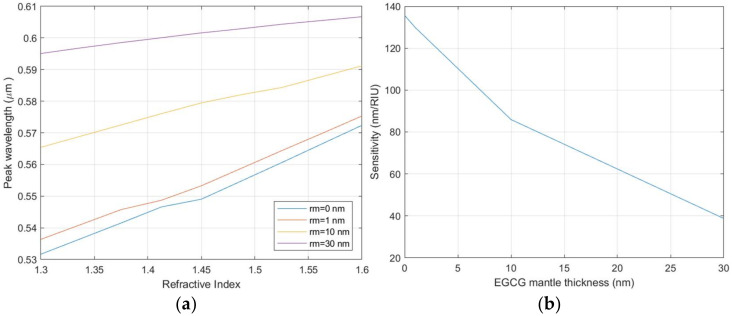
(**a**) Variation of the central wavelength for the LSPR resonance as a function of the medium refractive index for different thickness of the EGCG capping layer. (**b**) Sensitivity of the NPs’ LSPR as a function of the EGCG capping layer thickness.

Figure 11 reports the position of the LSPR central wavelength as a function of the refractive index of the surrounding medium and the corresponding sensitivity. The presence of the EGCG capping layer also acts as a separation layer between the AuNPs and the surrounding medium, reducing the sensitivity of the AuNPs. The simulation results show that when the thickness of the EGCG layer remains below a few tenths of a nanometer, even if reduced, the sensitivity value is maintained within the limits described in the literature [62,63]. The variation of the refractive index also produces a modification in the LSPR peak intensity. The combined analysis of peak wavelength and intensity can be used to improve the sensor signal-to-noise ratio (SNR), allowing a higher tolerance to the sensitivity reduction introduced by the EGCG capping layer.

## 3. Materials and Methods

Chloroauric acid (HAuCl_4_·3H_2_O) was purchased from Sigma-Aldrich (Sigma-Aldrich, Munich, Germany); the black tea leaves (*Thea sinensis)* for the tea extract preparation used as a reducing agent for chloroauric acid was purchased from Pingo Doce (Pingo Doce, Portugal, tea brand—batch 1832); graphene [64] and derivatives were synthetized using the modified Hummers method [64].

**Figure 12:**
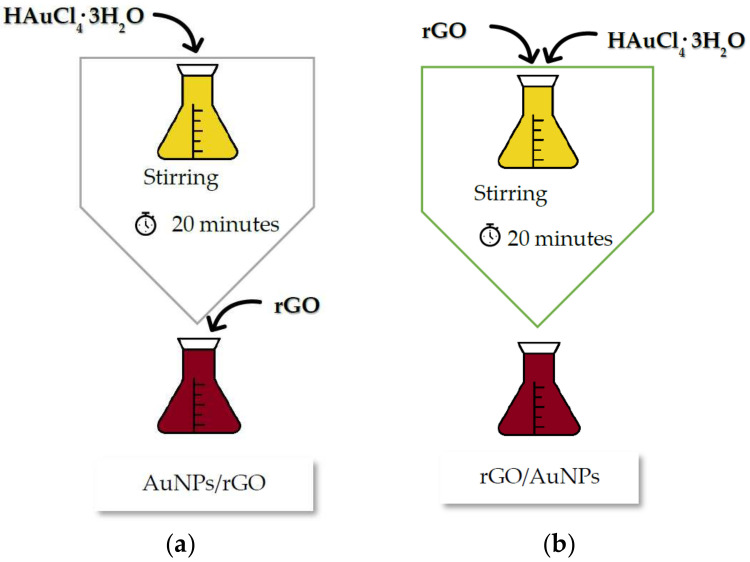
Preparation of samples by SQ1-AuNPs/rGO (**a**) or SQ2-rGO/AuNPs (**b**).

Black tea extracts with 5% concentration were prepared as reported previously [10], the main difference being that the resides used in the addition of graphene and its derivatives were added in a different order to see if this would produce different UV spectra (different transitions). This led to the following sequences (Figure 12):

Sequence 1 (SQ1): 1 mL of chloroauric acid (0.1 M) was added to each tea extract *Thea sinensis* concentration (5%) with a 6 mL total volume. This mixture was stirred for 20 min at 400 rpm at room temperature. After that, 1 mg of graphene or derivative was added to every 2 mL of the samples previously obtained (Figure 12a).

Sequence 2 (SQ2): The same weight of graphene or derivatives was added before the addition of chloroauric acid (Figure 12b). The remaining synthesis process of nanoparticles was maintained as described in SQ1.

Both nanoparticles and nanocomposites were characterized by UV–Vis spectroscopy (UV-2501PC Schimadzu, Waltham, MA, USA) and transmission electron microscopy (Hitachi 8100, Tokyo, Japan) with a ThermoNoran EDS light and zeta potential (Litesizer 500—Anton-Paar, Gratz, Austria). The characterization by UV–Vis was undertaken at 3 distinct moments, t_0_, t_1w_ and t_2w_, to determine the stability of the AuNPs and the nanocomposites over time, t_0_ being the starting moment, immediately after stirring the samples, t_1w_ one week after, and t_2w_ two weeks after. All samples were preserved in cold conditions and protected from light. TEM images were obtained between t_0_ and t_1w,_ as was the zeta potential of the samples.

Modified and unmodified graphene were analyzed by X-ray photoelectron spectroscopy with a XSAM800 spectrometer from KRATOS. Non-monochromatic radiation from a Mg Kα source was used (hν = 1253.6 eV). Powdered samples were fixed on the XPS holder with a double face tape and analyzed at UHV, at TOA = 45°. The BE was corrected considering the charge shift observed for the sp^2^ C-C and C-H peak set at 284.4 eV [52]. Other operational conditions and data treatment details were as published elsewhere [65].

## 4. Conclusions

The black tea extract (*Thea sinensis*) showed reducing capacity of chloroauric acid, allowing the synthesis of spherical gold nanoparticles. The production of AuNPs through this green synthetic approach proved to be sustainable, not only due to its low cost but also because of the reducing capacity of the tea and its coating agent function, conferring stability to the synthesized AuNPs. We found that 2 weeks after stirring of the reagents, polyphenols prevailed as coating agents, sustaining the stability of the AuNPs. Simulation results based on the Mie theory for the LSPR effects support the conclusion that, even with a thin capping layer of residuals, no significant reduction of plasmonic resonance should be expected.

The addition of carbon-based nanomaterials before stirring of the tea extract with chloroauric acid proved not to be efficient with respect to the stability of synthesized AuNPs, and, in some cases, LSPR did not take place at any of the moments of characterization. The reverse order of material addition, i.e., the addition of carbon-based nanomaterials after the stirring of the tea extract with metallic salt, proved to be more efficient, both in terms of the synthesis and stability of the AuNPs.

Of the three materials studied, rGO proved to be the most efficient carbon-based nanomaterial used as a support for AuNPs to be applied in biosensors. The stability revealed by the zeta potential, the greater dispersion of AuNPs, and the conductivity of this nanomaterial reported by several authors support this statement. Khalil et al. [6] corroborate our conclusion by stating that rGO has advantages as a support of AuNPs, since this conjugation enhances stability, inhibiting agglomeration through a closer contact between rGO and AuNPs, as also suggested by XPS results. The possibility of producing a metal–graphene hybrid nanostructure, composed of AuNPs and graphene allotropes, opens an interesting avenue for the exploration of biosensing applications, as these composites can be tuned to a specific wavelength of resonance, while at the same time they are known to simplify functionalization with bioelements (antibodies or antigens) for selective detection of specific biomarkers or disease carriers. Once joined to a low-cost optoelectronic setup for output extraction, a LSPR sensing element fabricated with these graphene–metal hybrid nanostructures (AuNPs-G-GO-rGO) could be of great use in a situation where a large-scale, low-cost, and timely disease screening action is needed, as, for example, in a future pandemic crisis or in Third World countries, where access to laboratory facilities is problematic.

The topic of this paper has not been widely explored in the literature and it will be necessary to carry out more exhaustive research in order to reach conclusions about certain questions raised in our study. Nevertheless, we believe that this work can be a good starting point for this investigation. Regarding future work, it would be interesting to monitor the synthesis of AuNPs between t_0_ and t_1w_ so that we can determine when this reaction ceases. In addition, trying other brands of black tea could supply additional valuable information, since different conditions, both soil and climatic, may be associated with different antioxidant properties.

## Figures and Tables

**Table 1:** Several applications of graphene and its derivatives-based optical sensors.

Optical Sensors	Material	Application	Ref.
Fluorescence sensing	GO	Effect of pH on fluorescence	[20]
Fluorescence sensing	GO	Fluorescence quencher	[21]
Fluorescence sensing	GO	Two-photon multi-color bio-imaging of multiple drug-resistant bacteria (MDRB)	[22]
Fluorescence sensing	GO	Fluorescence imaging	[23]
Fluorescence sensing	GO	High-sensitivity detection of miRNA in cells	[24]
Graphene-Based SERS Sensing	G	Adsorbed molecules	[25]
Graphene-Based SERS Sensing	G	Detection of biomarkers and biomolecules	[26]
Graphene-Based SERS Sensing	G	Bio-imaging, cancer diagnostics	[27]
Graphene-Based SERS Sensing	GO	Effects of pH values on SERS intensities of some aromatic molecules	[28]
Graphene-Based SERS Sensing	RGO	SERS effects of RGO with different degrees of reduction	[29]
Graphene-Based Optical Fiber Sensing	G	Biochemical sensing	[30]
Graphene-Based Optical Fiber Sensing	G	Gas sensor	[31]
Graphene-Based Optical Fiber Sensing	G	Biomolecule detector	[32]
Graphene-Based Optical Fiber Sensing	GO and RGO	Sensors for volatile organic compounds	[33]
Other Kind of Graphene-Based Optical Sensors	G	Detection of cancer cells	[34]
Other Kind of Graphene-Based Optical Sensors	RGO	Detection of cancer cells	[35]
Other Kind of Graphene-Based Optical Sensors	G and RGO	Photothermal detection (PTD)	[36]

**Table 2:** Zeta potential values of AuNPs synthesized before and after rGO addition.

Sample	Zeta Potential (mV)	Standard Deviation (mV)
5%_AuNPs	−15.59	0.595
5%_AuNPs/rGO	−20.17	0.868

**Table 3:** Corrected BE ± 0.1 eV and corresponding assignments.

	AuNPs/ rGO	AuNPs/ GO	AuNPs/ G	rGO	GO	G	Assignments [51,52]
C 1s	284.4	284.4	284.4	284.4	284.4 (^1^)	284.4	C-C and C-H sp^2^
	285.5	285.1	285.7	285.4	285.0	285.2	C-C and C-H sp^3^
	286.3	287.0	286.7	286.4	287.3	286.2	C-O or epoxide
	287.6	288.3	287.9	287.7		287.4	C=O
	288.8		289.1	288.8	289.2	288.8	XO-C=O (X=H or C)
	290.2	289.7	290.3	289.9		290.1	π-π*
	291.3		291.6	291.3	292.0	291.3	
	293.3		292.6	294.1		293.8	
	295.6		294.9				
O 1s	531.5	531.6	530.7	531.3			O in electropositive vicinity
	532.7	532.9	532.3	532.9	532.9	532.3	O bonded to C
Au 4f_7/2_	84.1	84.6	84.1				Au^0^; in “AuNPs/GO”: Au^+^? (see text)
Au 4f_5/2_	87.8	88.3	87.8				
S 2p_3/2_		168.6			169.0		SO_4_^2−^
S 2p_1/2_		169.8			170.0		
Si 2p_3/2_	101.7	101.7		101.6	102.0		silicone
Si 2p_1/2_	102.3	102.4		102.2	102.6		

(^1^) see text.

**Table 4:** XPS atomic concentrations (%) and relevant atomic ratios.

	AuNPs/ rGO	AuNPs/ GO	AuNPs/ G	rGO	GO	G
Atomic Concentrations (%)						
C	86.2	66.5	92.8	87.2	71.2	93.9
O	13.4	32.3	7.1	12.6	27.3	6.1
Au	0.12	0.03	0.06			
S		0.8			1.0	
Si	0.2	0.4		0.2	0.6	
Atomic ratios						
Au/C	0.0014	0.0004	0.0006			
O/C	0.15	0.44	0.08	0.14	0.33	0.06

## Supplementary Materials

The following supporting information can be downloaded at: https://www.mdpi.com/article/10.3390/bios12030163/s1: Figure S1: Frequency vs. size distribution: (a) for Figure 3a; (b) for Figure 3b and (c) for Figure 5; Figure S2: C 1s spectral differences between (a) AuNPs/rGO and rGO; (b) AuNPs/G and G and (c) AuNPs/GO and GO; Figure S3: TEM image for (a)—G; (b)—rGO and (c)—GO; Figure S4: LSPR of AuNPs and G composite (SQ1 and SQ2) at t_0_, t_1w_ and t_2w_; Figure S5: LSPR of AuNPs and GO composite (SQ1 and SQ2) at t_0_, t_1w_ and t_2w_; Figure S6: LSPR of AuNPs and rGO composite (SQ1 and SQ2) at t_0_, t_1w_ and t_2w_.
