# Supplementary material for: Effect of Graphene vs. Reduced Graphene Oxide in Gold Nanoparticles for Optical Biosensors—A Comparative Study
Source: Biosensors (Basel). 2022 Mar 4;12(3):163. doi: 10.3390/bios12030163 (PMC8946507; doi:10.3390/bios12030163)
Supplement: Supplementary file 1 [file biosensors-12-00163-s001.zip › biosensors-1587025-SI.pdf]

biosensors

MDPI

Supplementary Materials

Effect of Graphene vs. Reduced Graphene Oxide in Gold Nanoparticles for Optical Biosensors—A Comparative Study

Ana P. G. Carvalho [1,*], Elisabete C. B. A. Alegria [1,2], Alessandro Fantoni [3,4], Ana M. Ferraria [5,6], Ana M. Botelho do Rego [5,6] and Ana P. C. Ribeiro [2]

[1] Departamento de Engenharia Química, ISEL, Instituto Politécnico de Lisboa, 1949-014 Lisbon, Portugal; elisabete.alegria@isel.pt
[2] Centro de Química Estrutural, Instituto Superior Técnico, Universidade de Lisboa, 1049-001 Lisbon, Portugal; apribeiro@tecnico.ulisboa.pt
[3] Departamento de Engenharia Eletrónica e Telecomunicações e de Computadores, ISEL, Instituto Politécnico de Lisboa, 1949-014 Lisbon, Portugal; afantoni@deetc.isel.ipl.pt
[4] Centro de Tecnologias e Sistemas, UNINOVA, Faculdade de Ciências e Tecnologia, 2829-517 Caparica, Portugal
[5] iBB—Institute for Bioengineering and Biosciences and Departamento de Engenharia Química, Instituto Superior Técnico, Universidade de Lisboa, Av. Rovisco Pais, 1049-001 Lisbon, Portugal; ana.ferraria@tecnico.ulisboa.pt (A.M.F.); amrego@tecnico.ulisboa.pt (A.M.B.d.R.)
[6] Associate Laboratory i4HB—Institute for Health and Bioeconomy at Instituto Superior Técnico, Universidade de Lisboa, Av. Rovisco Pais, 1049-001 Lisbon, Portugal
* Correspondence: ana.carvalho.ana@gmail.com

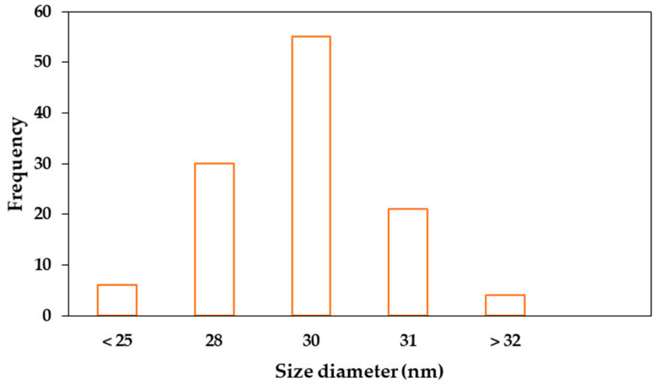

Copyright: © 2022 by the authors. Licensee MDPI, Basel, Switzerland. This article is an open access article distributed under the terms and conditions of the Creative Commons Attribution (CC BY) license (https://creativecommons.org/licenses/by/4.0/).

(a)

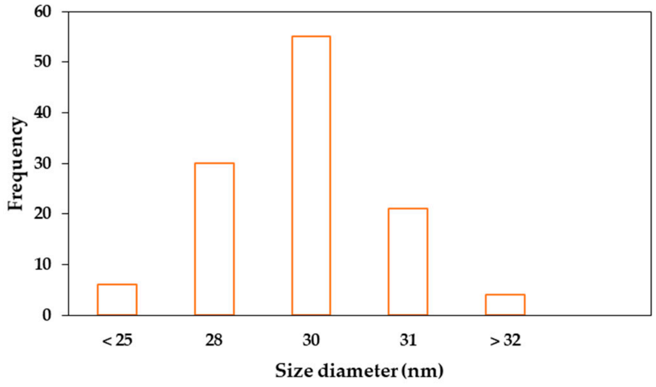

(b)

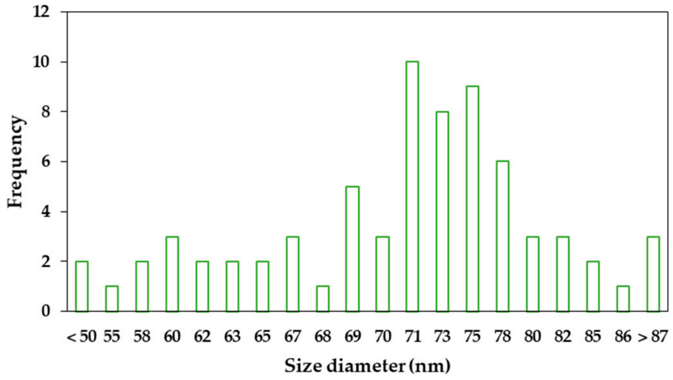

(c)

Figure S1. Frequency vs. size distribution: **a**) for Figure 3a; **b**) for Figure 3b and **c**) for Figure 5.

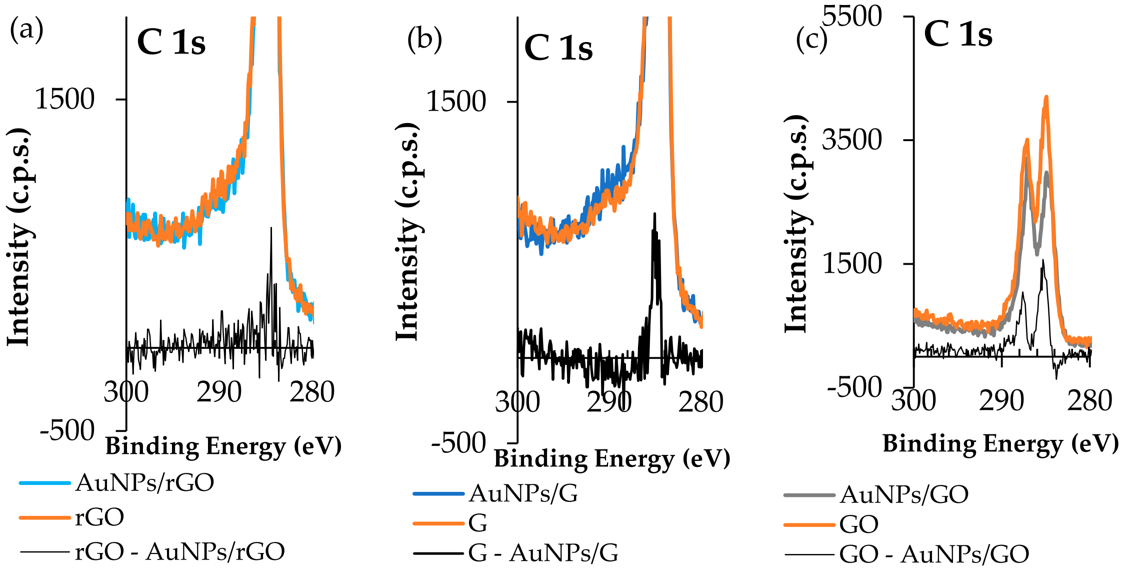

(a) **(b)** **(c)**

Figure S2. C 1s spectral differences between(**a**) AuNPs/rGO and rGO; (**b**) AuNPs/G and G and (**c**) AuNPs/GO and GO.

Figure S2 shows that: rGO has slightly more sp[2] carbon than AuNPs/rGO; G has a larger amount of aliphatic carbon than AuNPs/G. In AuNPs/GO versus GO, one can see that the C 1s signal for GO is stronger than for AuNPs/GO, which leads to a spectrum "difference" showing more aliphatic carbon and more oxidized carbonaceous moieties in GO. However, it is clear from the C 1s profiles that in GO, the main peak, centred at lower BE (which includes peaks at 284.4 eV and 285 eV, attributed to non-oxidized carbon atoms), is larger than the peak at higher BE, which includes carbon atoms bonded to oxygen (Figure 6 (d)). In AuNPs/GO, the oxidized carbon features have nearly the same intensity as the non-oxidized carbon peak.

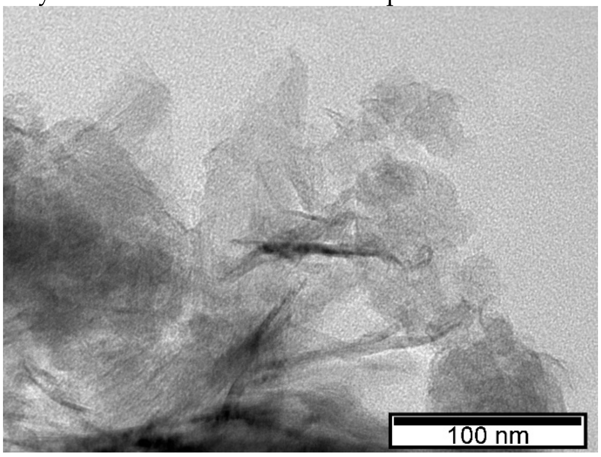

(a)

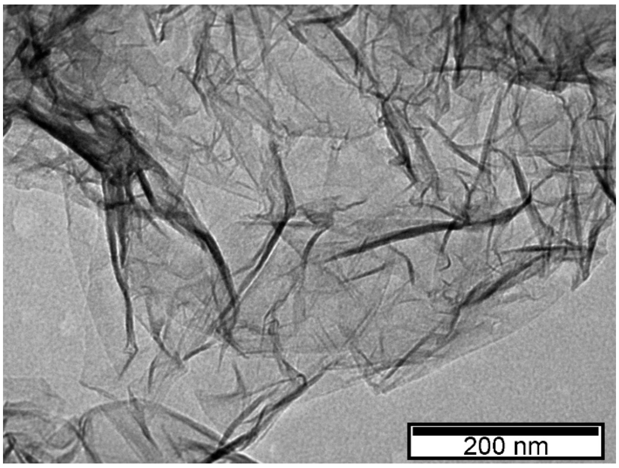

(b)

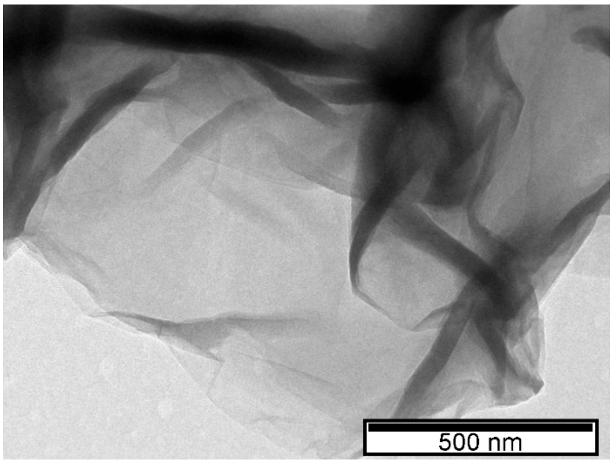

(c)

Figure S3. TEM image for (**a**) – G; (**b**) – rGO and (**c**) – GO.

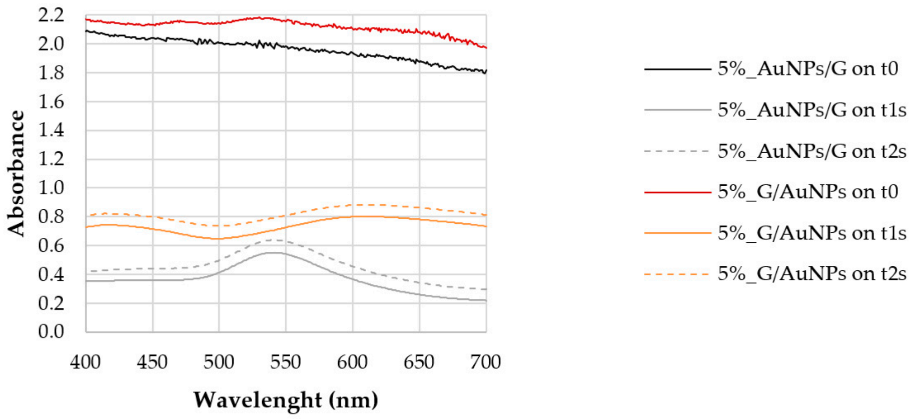

Figure S4. LSPR of AuNPs and G composite (SQ1 and SQ2) at t_0, t_{1w} and t_{2w}.

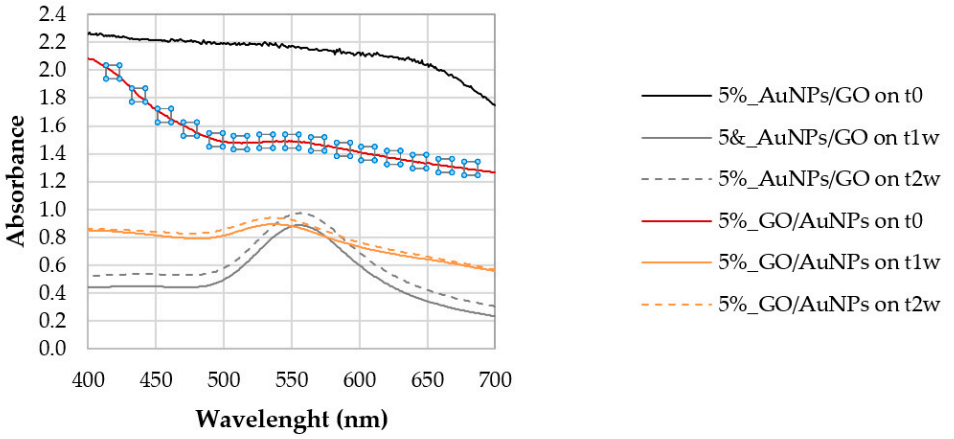

Figure S5. LSPR of AuNPs and GO composite (SQ1 and SQ2) at t_0, t_{1w} and t_{2w}.

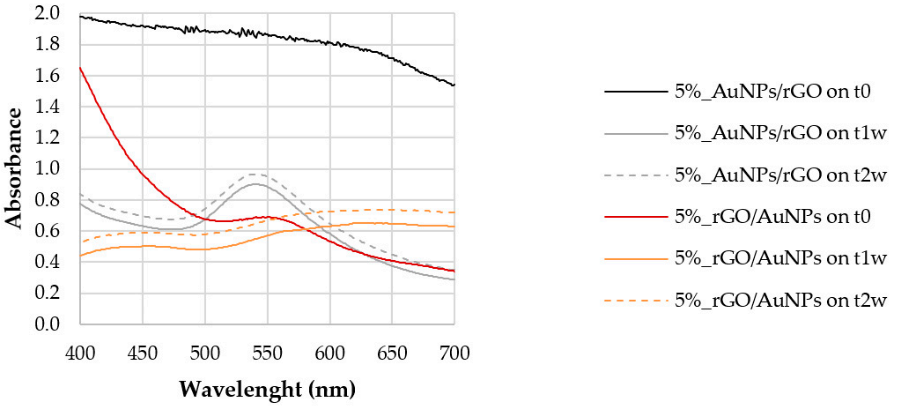

Figure S6. LSPR of AuNPs and rGO composite (SQ1 and SQ2) at t_0, t_{1w} and t_{2w}.
